# Assessing cultural awareness and competency in nursing education: A cross-sectional study of undergraduate nursing students in Ireland

**DOI:** 10.1371/journal.pone.0359851

**Published:** 2026-10-12

**Authors:** Jennifer Sullivan, Sahar Hammoud

**Affiliations:** 1 University of Galway, Galway, Ireland; 2 School of Nursing, Midwifery and Health Systems, Health Sciences Centre, University College Dublin, Dublin, Ireland; 3 UCD Centre for Interdisciplinary Research, Education and Innovation in Health Systems (UCD IRIS), School of Nursing, Midwifery and Health Systems, Health Sciences Centre, University College Dublin, Dublin, Ireland; Al Azhar University Gaza, PALESTINE, STATE OF

## Abstract

**Background:**

There is a growing imperative for nurses to navigate multicultural healthcare systems and deliver quality, responsive, safe, person-centred care. Accordingly, undergraduate nursing education must be relevant and innovative to provide undergraduate nursing students with meaningful learning opportunities to develop cultural competence. This study aims to assess undergraduate nursing students’ cultural awareness and examine how these perceptions vary according to socio-demographic characteristics.

**Methods:**

A descriptive cross-sectional study was conducted utilising a valid and standardised tool, the cultural awareness scale (CAS). “One hundred and sixteen” undergraduate nursing students from a higher education institution participated in the study. The Mann–Whitney U test and the Kruskal–Wallis test were used to examine differences in CAS scores across subscales and total scores according to participants’ socio-demographic characteristics.

**Results:**

Overall, the CAS results showed students’ level of cultural awareness was moderately high, with slight variations across the four CAS subscales. Participants born in Ireland had higher scores on all subscales compared to participants born outside Ireland, although these differences were not statistically significant. Participants who identified as White scored highest on General Education Experience (64.54 vs 42.66, U = 837, *p = 0.002, r = −0.29*), and Total Awareness (63.32 vs 45.86, U = 939.5, *p = 0.012, r = −0.23*) compared with participants who were categorised as people of colour.

**Conclusions:**

The cultural awareness scores in this study were moderately high among undergraduate nursing students, consistent with previous research using the CAS instrument. The findings suggest an association between ethnic background and perceptions of culturally inclusive educational practices and highlight the need for further research to explore these patterns and inform the development of culturally responsive nursing education.

## Background

Ireland has seen rapid expansion and population growth; by 2030 it is projected that 18% of the population will be born outside of Ireland, up from 10.4% in 2023 [1]. This rapid expansion has led to a multicultural healthcare community spanning patients, healthcare providers (54% of practising nurses and 41.3% of practising physicians in Ireland in 2024 were internationally trained) [2] and healthcare students. The younger population in Ireland reflects a steadily expanding diversity of national origins, cultural identities and sociodemographic backgrounds [3]. The surge in immigration and expanding socioeconomic inequality has highlighted significant gaps in our understanding of diversity and inclusion, especially in the provision of inclusive healthcare and education [4].

As populations become increasingly diverse, the disease risk profile of majority populations often differs from those of ethnic minority populations [5]. Ethnic minority populations show significant inequalities in hospital admissions [6]. It is reported within Europe that underrepresented population subgroups experience worse quality care than the general population [7]. Population groups with differences determined by socio-demographic backgrounds such as culture, religion or ethnicity show differences in terms of illness behaviour and beliefs when accessing services [8].

The World Health Organization [9] explains that people’s health needs, health beliefs and behaviours are shaped by culture, stating that behaviours are the single most influential factor determining people’s health. The Irish healthcare system recognises the need for healthcare staff to enhance their knowledge, skills and awareness in delivering and adapting to future population healthcare needs, leading to the development of the National Intercultural Health Strategy, which aims to provide quality, responsive, culturally competent healthcare [10] to service users from diverse ethnic, cultural and religious backgrounds [11]. Cultural competence is a continuous process in which the healthcare provider strives to effectively work within the cultural context of the patient. The process goes beyond knowledge acquisition and involves the integration of cultural awareness, cultural knowledge, cultural skill, cultural encounters and cultural desire into practice [10].

In Ireland, healthcare quality is defined by the four quality domains outlined in the National Standards for Safer Better Healthcare [12]. One of these four quality domains is person-centred care (PCC), which is characterised by care that is respectful and responsive to an individual’s needs, values, preferences and diversity. The PCC approach to care involves partnering with the individual in the design and delivery of their care, ensuring non-discrimination based on age, gender, sexual orientation, disability status, civil or marital status, race, religious beliefs, or membership of the Traveller community [13].

Nurses are professionally obliged to appropriately address the diverse, unique cultural healthcare needs of all people in their care across the life span, including sexual health, women’s and men’s health, mental health, reproductive health, palliative care, the healthcare needs of children and young people, LGBTQIA+ individuals, and people with a disability [14]. There is evidence to suggest that cultural factors influence individuals’ health beliefs and health behaviours; understanding health behaviours reduces barriers and makes healthcare relevant and accessible to all populations [15]. Cultural competence is an evolving process, and the process begins with cultural awareness, which is the self-examination of one’s own culture and background, and the recognition of one’s biases, prejudices and assumptions about individuals who are different [10]. It serves as the foundation for all healthcare providers in the delivery of safe, effective PCC.

Given there is evidence to develop nurses’ cultural competence in undergraduate nursing education, there is a lack of consensus in standardising educational interventions and the success rates of cultural competence interventions [16]. Markey [17] reports deficits in cultural knowledge and a lack of confidence in sensitively addressing the care needs of culturally diverse service users as challenges to the readiness of nurses in providing culturally competent practice. The Nursing and Midwifery Board of Ireland’s (NMBI) recent national curriculum review recognises the challenges to future-proofing undergraduate nursing and midwifery student preparedness for the healthcare needs of a diverse patient population that reflects contemporary healthcare delivery [18].

The Cultural Awareness Scale (CAS) has been applied in undergraduate nursing programmes internationally, including studies in the United States of America, Europe and Asia [19–24], where it has primarily been used to validate the instrument and describe students’ cultural awareness levels. These studies consistently report moderate to high cultural awareness scores, but they do not examine cultural awareness within an Irish context or explore socio-demographic variation in an Irish nursing student population. Therefore, there is a clear need for Irish nursing programmes to measure cultural awareness among undergraduate nursing students in higher education. This study addresses this gap by assessing undergraduate nursing students’ cultural awareness – an early stage of cultural competence development – and examining how these perceptions vary according to socio-demographic characteristics. In doing so, it builds on existing CAS-based research by providing new evidence from an Irish setting and by comparing the CAS scores observed in this cohort with those reported in previous international studies.

## Materials and methods

### Study design and setting

A descriptive, cross-sectional study was conducted during the academic year 2022–2023 in a higher education institution in Ireland. This study was conducted and reported in accordance with the Strengthening the Reporting of Observational Studies in Epidemiology (STROBE) guidelines [25]. The completed checklist is provided in S1 Appendix.

### Study sample and eligibility criteria

Purposive sampling was used to enrol participants. All participants who met the following criteria were eligible to participate: a third- or fourth-year undergraduate nursing student, enrolled in a four-year bachelor’s programme in general nursing or children’s and general nursing, and registered for a general module. Purposive sampling was used because the study aimed to assess cultural awareness among nursing students who had sufficient academic and clinical exposure to meaningfully reflect on cultural encounters. Students in years three and four have completed substantial theoretical modules and multiple clinical placements, providing the experiential foundation required to engage with the subscales measured by the used scale. Year one and year two students were excluded because they have limited clinical exposure and may not yet have encountered culturally diverse situations in practice, making their inclusion less aligned with the study aim.

A formal sample size calculation was not conducted, as the study aimed to survey the entire population of eligible undergraduate nursing students in years three and four. All students who met the inclusion criteria (N = 239) were invited to participate, and 116 consented to take part. The final sample therefore reflects the maximum achievable number of respondents from this defined population, resulting in a response rate of 48.5%.

### Instrument and variables

The CAS tool was utilised as a pre-existing instrument developed and validated by Rew et al. [19] to assess cultural awareness among healthcare professionals and student nurses. It is a recognised and reliable tool that has been employed in previous studies [20]. The original CAS is a 36-item Likert-type questionnaire comprising five domains: General Education Experience (GEE), Cognitive Awareness (CA), Behaviours/Comfort with Interactions (BCI), Research Issues (RI), and Patient Care/Clinical Issues (PCCI). Each item is rated on a 7-point scale ranging from 1 (strongly disagree) to 7 (strongly agree).

To ensure relevance to the Irish undergraduate nursing context, the scale underwent a face-validity review by five expert reviewers (three senior nursing lecturers and two clinical tutors). Minor wording adjustments were recommended to reflect Irish terminology and institutional context. Although the term *instructor* is not commonly used in Ireland, it was retained in the questionnaire; however, a definition was provided at the beginning of the scale to support clarity for participants. *Instructor* was defined as “a teacher of a college or university subject; a person whose job is to teach people a skill” [26]. In addition, the definition of *cultural awareness* was provided as “awareness of our own cultural identity, values, and beliefs, and the knowledge and acceptance of others’ cultures” [27].

The RI domain was excluded because its items relate to research engagement and decision-making processes that are not applicable to undergraduate nursing students, who typically have limited research experience and, at this stage of their programme, have not yet completed thesis or dissertation work. Following removal of the RI domain, the final instrument used in this study comprised 32 items across four domains. The total CAS score ranged from 32 to 224, with higher scores indicating greater cultural awareness. After these minor revisions, the expert reviewers reassessed the scale and confirmed face validity, with no further changes required.

The original CAS demonstrated good internal consistency (Cronbach’s α = 0.82) in its validation study by Rew et al. [19]. In this study, a full psychometric validation of the modified CAS was not feasible due to resource constraints, as the project formed part of the first author’s Master’s research. However, internal consistency reliability was assessed. The overall modified scale demonstrated good reliability (Cronbach’s α = 0.75). Subscale reliability coefficients ranged from good to low: GEE (α = 0.75), PCCI (α = 0.66), CA (α = 0.59), and BCI (α = 0.55). Notably, the lower reliability of the BCI subscale is consistent with findings reported in previous studies conducted in Poland, South Korea, and in the original CAS development study [19,24,28]. Cronbach’s alpha is influenced by the number of items in a scale, and shorter subscales typically yield lower alpha values [29]; therefore, the relatively low reliability coefficients for the BCI and CA domains are not unexpected given their limited number of items.

These reliability results provide evidence that the modified 32-item CAS performed adequately in this sample, although the removal of the RI domain may have influenced the overall psychometric properties of the instrument. The four domains were as follows:

Domain 1 (GEE): Perception of cultural awareness application in education (14 items; score range 14–98).
Domain 2 (CA): Cognitive Awareness of the impact of cultural diversity on own attitudes and behaviours (7 items; score range 7–49).
Domain 3 (BCI): Comfort while supporting people from diverse groups (6 items; score range 6–42).
Domain 4 (PCCI): Application of cultural competency in practice (5 items; score range 5–35).

For this study the Central Statistics Office Data Standard for Ethnicity classification was used to capture the demographic multicultural characteristics of participants [30]. Categories included Black background (Black, Black Irish, Other), White background (White, White Irish, Other White), Asian background (Asian, Asian Irish, Other) and Other (Arab, Other).

### Data collection procedure

Written informed consent was obtained through a participant information sheet outlining the study’s purpose, procedures, consent process and measures, to ensure anonymity and confidentiality.

Data collection took place in classrooms at the School of Nursing, Midwifery and Health Systems of a higher education institution in Ireland, in November 2022 and January 2023. At the start of each session, students were provided with verbal and written information about the voluntary and anonymous nature of the study, with an opportunity to ask questions.

Paper surveys and a sealed collection box were then made available. The researcher left the classroom to allow students to complete the survey privately and return it to the sealed box. The box was subsequently collected by a third party. A total of 116 fully completed questionnaires were included in the final analysis.

### Data analysis

Data were managed and analysed using IBM SPSS Statistics (version 29). Descriptive statistics were calculated as frequencies and percentages for categorical variables, and as means with standard deviations (±SD) for continuous variables. The Shapiro–Wilk test was used to assess the normality of the data, which indicated that the data were not normally distributed. Consequently, non-parametric tests were applied: the Mann–Whitney U test and the Kruskal–Wallis test were used to examine differences in cultural awareness scores across subscales and total scores according to participants’ demographic characteristics. Statistical significance was set at p < 0.05. Missing data were minimal, and no surveys contained more than 20% missing responses; therefore, all completed questionnaires were retained for analysis.

Ethnicity was originally collected using six categories (White (n = 84), Asian (n = 8), Asian Irish (n = 3), Black (n = 6), Black Irish (n = 11), and Other; Arab/Other (n = 4)). Due to small subgroup sizes across the Asian, Asian Irish, Black, Black Irish, and Other categories, statistical comparisons using the original categories were not feasible. To ensure adequate cell sizes and maintain the validity of non‑parametric tests, these categories were collapsed into a binary variable comprising “White” (n = 84) and “People of Colour” (n = 32). This approach is commonly used in quantitative analyses with small subgroup counts, although it necessarily combines heterogeneous populations. The implications of this decision are acknowledged in the discussion section.

In addition to *p* values, effect sizes were calculated for all tests (r for Mann–Whitney U and η² for Kruskal–Wallis) to aid interpretation of the magnitude of observed differences. Multivariate analysis, such as multiple regression or ordinal regression, was considered; however, the overall sample size and small subgroup counts – particularly within the ethnicity variable (n = 32 for people of colour) – limited the feasibility of such models and would likely result in unstable parameter estimates. Therefore, analyses were restricted to non-parametric bivariate tests.

### Ethical approval

The study was conducted in accordance with the principles of the Declaration of Helsinki and received ethical approval from the University College Dublin Life Sciences Research Ethics Committee (Reference number: LS-C-22-234-Sullivan). All participants provided written informed consent before taking part. Detailed information about the study was provided, and participation was entirely voluntary.

## Results

### Demographic characteristics of participants

Of the 116 participants, the majority identified as women (93.1%), identified as White (72.4%), born in Ireland (79.1%), and aged between 18 and 24 years (86.2%). Full details of the participants’ demographic characteristics are presented in Table 1.

**Table 1:** Demographic characteristics of study participants (n = 116).

Demographic	Number of participants (%)
**Gender**	
Woman	108 (93.1%)
Man	7 (6%)
Nonbinary	1 (0.9%)
Total	116
**Age**	
18–24	100 (86.2%)
25—34	10 (8.6%)
>34	6 (5.2%)
Total	116
**Place of birth**	
Ireland	91 (79.1%)
Outside Ireland	24 (20.9%)
Total	115
**Parents’ place of birth**	
Both in Ireland	63 (54.3%)
Both outside Ireland	40 (34.5%)
One in Ireland, one outside Ireland	12 (10.3%)
I don’t know	1 (0.9%)
Total	116
**Ethnicity**	
White participants	84 (72.4%)
Person of colour participants	32 (27.6%)
Total	116

### Cultural awareness scores

The results for the individual items of the CAS subscales are presented in S1 Table.

The mean scores for each CAS subscale, as well as the overall CAS score, are presented in Table 2.

**Table 2:** Mean scores of Cultural Awareness Scale among participants (n = 116).

Scale/Subscale	Mean ± Standard Deviation
General Education Experience (GEE)	67.71 ± 9.54
Cognitive Awareness (CA)	37.29 ± 4.41
Behaviours/Comfort with Interactions (BCI)	34.21 ± 4.66
Patient Care/Clinical Issues (PCCI)	27.56 ± 3.99
Total Awareness	173.32 ± 14.93

### Cultural awareness scores across demographics

Table 3 presents the cultural awareness scores for each subscale and the total CAS scores across participants’ demographic characteristics. No statistically significant differences in awareness scores were observed across gender, age, or parents’ place of birth. Participants born in Ireland demonstrated higher awareness scores across all subscales compared with those born outside Ireland; however, these differences were not statistically significant.

**Table 3:** Cultural Awareness Scale scores across demographics (n = 116).

	GEE	CA	BCI	PCCI	Total Awareness
Demographic	Mean Rank				
**Gender** ^**‡**^					
Woman	57.96	58.89	59.37	58.50	58.60
Man	66.64	48.71	40.07	51.71	51.21
Nonbinary	60.00	84.50	93.50	105.50	99.00
***p-value***	*0.802*	*0.545*	*0.194*	*0.323*	*0.410*
***η²***	*0.00*	*0.01*	*0.03*	*0.02*	*0.02*
**Age** ^**‡**^					
18–24	58.64	60.73	59.55	57.42	59.49
25–34	52.05	50.25	62.20	68.10	56.20
>34	66.92	35.08	34.83	60.50	45.83
***p-value***	*0.688*	*0.137*	*0.201*	*0.623*	*0.611*
***η²***	*0.01*	*0.03*	*0.03*	*0.01*	*0.01*
**Place of birth** ^**†**^					
Ireland	59.04	59.52	59.46	58.74	60.04
Outside Ireland	54.06	52.23	52.48	55.21	50.27
***p-value***	*0.515*	*0.339*	*0.360*	*0.643*	*0.201*
***r***	*−0.06*	*−0.09*	*−0.09*	*−0.04*	*−0.12*
**Parents’ place of birth** ^**‡**^					
Both in Ireland	64.12	53.75	60.62	60.94	63.04
Both outside Ireland	49.35	67.94	53.70	59.75	52.40
One in Ireland, one outside Ireland	57.17	49.79	67.08	38.46	53.54
I don’t know	86.50	84.50	14.00	95.00	76.00
***p-value***	*0.142*	*0.119*	*0.305*	*0.118*	*0.392*
***η²***	*0.05*	*0.05*	*0.03*	*0.05*	*0.03*
**Ethnicity** ^**†**^					
White participant	64.54	56.18	61.72	58.78	63.32
Person of colour participant	42.66	64.59	50.05	57.77	45.86
***p-value***	***0.002***	*0.227*	*0.884*	*0.094*	***0.012***
**r**	*−0.29*	*−0.11*	*−0.16*	*−0.01*	*−0.23*

†Mann-Whitney U test and ^‡^Kruskal-Wallis test were used for the comparison. Effect sizes are reported as *r* for Mann–Whitney U tests and *η²* for Kruskal–Wallis tests. Significant *p‑*values are presented in bold.

GEE = General Education Experience; CA = Cognitive Awareness; BCI = Behaviours/Comfort with Interactions; PCCI = Patient Care/Clinical Issues.

Participants who identified as White had higher awareness scores in the GEE, BCI, and PCCI subscales, as well as higher total CAS scores, compared with participants who were categorised as people of colour. These differences were statistically significant for GEE (64.54 vs 42.66, U = 837, *p = 0.002*, *r = −0.29*) and for Total Awareness (63.32 vs 45.86, U = 939.5, *p = 0.012, r = −0.23*). The effect sizes for both comparisons were small-to-moderate (*r = −0.29* for GEE; *r = −0.23*for Total Awareness), indicating that although statistically significant, the magnitude of the differences was modest.

## Discussion

Culturally responsive care is essential in nursing education, an expected professional requirement outlined by the NMBI to ensure healthcare delivery is safe, respectful, non-discriminatory and person-centred [31]. This study aimed to assess undergraduate nursing students’ cultural awareness and examine how these perceptions vary according to socio-demographic characteristics. The overall cultural awareness scores were moderately high across the four subscales, suggesting generally positive perceptions of cultural awareness among nursing students. These findings are consistent with previous studies using the CAS [20–22].

Participants born in Ireland scored higher on the CAS domains compared with those born outside Ireland; however, these differences were not statistically significant. Given the lower reliability of the CA and BCI subscales, these non‑significant results should be interpreted cautiously, as limited measurement precision may obscure true differences. This pattern may imply that students recognise and navigate cultural differences to varying degrees, potentially shaped by their backgrounds, experiences, or social environments. While these differences were not statistically significant, they highlight areas where perceptions of cultural awareness vary within the student population. Participants aged 18–24 years scored higher on the total CAS scores, while those aged >34 years scored the lowest, indicating a pattern of variation across age groups. Although these associations were not statistically significant, they highlight demographic patterns that may warrant further exploration in future research with larger and more diverse samples. Most recent Higher Education Authority (HEA) demographic trends indicate rising enrolments of mature, international, and non-EU students are key drivers of increasing diversity and expansion within the higher education sector [32].

Participants’ scores across the four subscales were moderately high, with slightly lower scores observed on the GEE and CA domains. Future studies conducted across multiple time points and academic years would better capture students’ education experiences and provide a more robust understanding of cultural awareness development. Understanding the composition and diversity of the student community remains essential for informing best practices in equity, diversity, and inclusion (EDI) within nursing education, particularly as Irish higher education institutions continue to diversify [33]. These patterns, although not statistically significant, underscore the importance of monitoring demographic variations in cultural awareness as part of ongoing EDI efforts in nursing education and warrants further investigation.

Despite ongoing efforts to promote cultural awareness within the higher education institution through EDI strategy actions and initiatives, the findings indicate an association between students’ ethnic background and their perceptions of culturally inclusive educational practices. Participants who were categorised as people of colour reported lower scores on the GEE subscale compared with those identified as White, suggesting that students’ experiences of general educational inclusivity may vary across demographic groups. Although this difference was statistically significant, the effect size was small‑to‑moderate (*r* = −0.29), indicating that the magnitude of the association was modest. A similar pattern was observed for the Total Awareness score (*r* = −0.23), where students identified as White scored higher; again, the effect size suggested a modest practical difference. These findings highlight subtle but persistent differences in perceptions of cultural inclusivity that warrant further exploration.

While the findings indicate an association between ethnicity and perceptions of cultural inclusivity, caution is warranted in interpreting these results. The binary grouping of “people of colour” necessarily combines individuals from diverse ethnic backgrounds, each of whom may have distinct cultural experiences and interactions within the educational environment. As such, the observed differences in GEE and Total Awareness scores may reflect broader differences in educational experience or institutional belonging rather than differences in cultural awareness per se. These nuances cannot be fully captured within a dichotomised variable, and the findings should not be generalised across all subpopulations included within the “people of colour” category. Future research with larger and more diverse samples, including mixed‑methods or qualitative designs, is needed to explore these subgroup differences in greater depth and to provide richer insight into how students from different ethnic backgrounds experience cultural inclusivity within nursing education. Additionally, the present findings relied on bivariate analyses, which do not adjust for potential confounding variables, due to the non-feasibility of multivariate analysis with the current sample and subgroup sizes. Future studies with larger samples should incorporate multivariate modelling to more fully account for confounding.

Existing literature highlights as student populations become increasingly diverse, universities have opportunities to strengthen culturally responsive learning experiences [34,35]. Within nursing education, long held values and traditions may create barriers to student success in contemporary classrooms [36], underscoring the vital role of nurse educators in modelling EDI competencies and fostering students’ sense of belonging [37]. Diversity within the nursing workforce is increasingly recognised as essential for communicating multiple perspectives and supporting PCC [38].

Evidence from the wider literature suggests that traditional cultural competence teaching approaches, such as guest speakers or study-abroad programmes, may not sufficiently support the development of culturally responsive behaviours [16,39], and relying on knowledge alone is not sustainable for cultural competence development in undergraduate nursing education [40]. The literature highlights complementary strategies to align with foundational knowledge, such as building on nurses leadership skills through critical consciousness to strengthen the ability to recognise and challenge healthcare inequality [41,42], and utilising role play and experiential learning to further scaffold and demonstrate cultural competence development [43]. Additional literature explores co-design as collaborative approach for inclusive curriculum development that reflects real-world healthcare needs [44], and the potential of healthcare simulation towards making cultural competence teaching and learning clinically relevant [45,46]. These approaches were not examined in this study but may offer useful avenues for enhancing cultural competence development within undergraduate nursing programmes.

### Implications for research and practice

This study provides initial insight into cultural awareness among undergraduate nursing students in Ireland and highlights areas where perceptions vary across socio-demographic groups. These findings suggest the importance of monitoring demographic patterns in cultural awareness and ensuring that educational practices support equitable learning experiences for all students. The moderately high awareness scores observed across the CAS domains indicate a positive foundation on which further development can be built, while the slightly lower scores on the GEE and CA domains point to areas where additional support may be beneficial.

Beyond these study-specific implications, evidence from the wider literature offers complementary strategies that may support inclusive nursing education. These include co-designing curricula with students, integrating patient and public involvement and incorporating simulation-based learning to enhance cultural responsiveness. While these approaches were not directly examined in this study, they may provide useful avenues for strengthening cultural competence development within undergraduate nursing programmes.

### Strengths and limitations

This study contributes to advancing cultural competency in nursing education by providing an initial exploration of cultural awareness among undergraduate nursing students in Ireland. The findings offer a foundation for future research and may inform the development of targeted educational strategies to support cultural competence within curriculum design.

Several limitations should be acknowledged. First, the sample size was relatively small, and data were collected from a single higher education institution, which limits the generalisability of the findings. Although the institution is one of the largest in Ireland and attracts students from diverse regions and international backgrounds, this may not ensure representativeness of all Irish nursing programmes. Student demographic profiles, curricular structures, and the extent to which cultural competency content is embedded can vary across institutions. Future research involving multiple higher education institutions nationally would provide a more comprehensive understanding of cultural competency education in Ireland and enhance the generalisability of findings. Second, the removal of the RI domain may have influenced the psychometric properties of the instrument. Cronbach’s alpha is sensitive to item count, and the lower reliability values observed for the shorter subscales (particularly BCI and CA) may reflect item reduction rather than conceptual weakness. The modified CAS may therefore differ in construct validity from the original instrument, and further psychometric evaluation within the Irish context is recommended. The exclusion of the RI domain also limits comparability with studies using the full 36 item CAS, although the four retained subscales remain comparable as all original items were preserved. Third, the timing of data collection – at the end of the first semester and the beginning of the second – may have influenced scores, particularly for the GEE subscale, which can vary depending on the amount of curriculum content students have been exposed to at different points in the academic year. Future studies conducted across multiple time points and academic years would help capture temporal variations in students’ experiences.

Additionally, several potential biases should be considered when interpreting the findings. First, the use of purposive sampling may introduce selection bias, as only students in years three and four were included. Although this aligns with the study aim, the sample may not fully represent all undergraduate nursing students. Furthermore, the sample was predominantly young, female, and White, which reflects the demographic profile of nursing programmes in Ireland but nonetheless may limits representativeness. These characteristics should be considered when interpreting the findings, as cultural awareness may vary across more diverse student populations. Second, non-response bias is possible, as students who chose not to participate may differ in their cultural awareness or comfort with the topic. We acknowledge that the response rate was moderate-to-low and that no data were collected on non-responders, therefore the direction or magnitude of any such bias cannot be determined. Third, the CAS is a self-reported instrument, which introduces the risk of self-report bias and, in particular, social desirability bias. Given the culturally sensitive nature of the topic, participants may have responded in ways they perceived to be socially acceptable or professionally expected rather than reflecting their actual attitudes or experiences. This may have influenced the magnitude of scores and the statistically significant differences observed between participants identified as White and those categorised as people of colour.

Finally, collapsing the ethnicity variable into a dichotomous grouping (“White” and “people of colour”) may mask important subgroup differences and limits the nuance of interpretation. The analyses also relied on non‑parametric bivariate tests, as multivariate analysis was not feasible given the overall sample size and small subgroup counts; therefore, potential confounding cannot be fully ruled out. The relatively small number of participants categorised as people of colour (n = 32) may have limited statistical power to detect subgroup differences, and the robustness of these findings should be interpreted cautiously. Future studies with larger and more diverse samples should incorporate multivariate analysis to more fully account for confounding.

## Conclusions

The cultural awareness scores in this study were moderately high across all four CAS subscales, consistent with previous research using the same instrument. Students identified as White scored significantly higher on the GEE subscale and on Total Awareness compared with students who were categorised as people of colour, although the small-to-moderate effect sizes indicate that these differences were modest. These findings suggest an association between ethnic background and perceptions of culturally inclusive educational practices and highlight areas where further exploration may be warranted.

As an initial examination of cultural awareness within an Irish undergraduate nursing context, this study provides a foundation for future research. Qualitative or mixed-methods studies would offer deeper insight into students’ experiences and help clarify the factors that shape perceptions of cultural inclusivity. Larger and more diverse samples, alongside multivariate approaches, will be important for strengthening the evidence base and informing the ongoing development of culturally responsive nursing education.

## Supporting information

S1 Appendix STROBE Checklist. S1 Appendix provides the completed STROBE checklist for this study.
(DOCX)

S1 Table Participants scores using the Cultural Awareness Scale (N = 116). S1 Table presents the results for the individual items of the CAS subscales.
(DOCX)
