# Supplementary material for: Assessing cultural awareness and competency in nursing education: A cross-sectional study of undergraduate nursing students in Ireland
Source: PLoS One. 2026 Oct 12;21(10):e0359851. doi: 10.1371/journal.pone.0359851 (PMC13657305; doi:10.1371/journal.pone.0359851)
Supplement: Supplementary file 2 [file pone.0359851.s002.docx]

Supplementary Material

**S2 Table.** Participants scores using the Cultural Awareness Scale (N= 116).

| Question | Number of participants (%) | | | | | | | |
| --- | --- | --- | --- | --- | --- | --- | --- | --- |
|  | Strongly disagree | Disagree | Somewhat disagree | Neither agree nor disagree | Somewhat agree | Agree | Strongly agree | Total |
| 1. The nursing instructors in the school of nursing midwifery and health systems (SNMHS) adequately address multicultural issues in nursing | 3 (2.6%) | 6 (5.2%) | 11 (9.6%) | 6 (5.2%) | 31 (27%) | 44 (38.3%) | 14 (12.2%) | 115 |
| 2. The SNMHS provides opportunities for activities related to multicultural affairs | 5 (4.3%) | 20 (17.2%) | 18 (15.5%) | 22 (19%) | 25 (21.6%) | 20 (17.2%) | 6 (5.2%) | 116 |
| 3. During my studies my understanding of multicultural issues has increased | 2 (1.7%) | 10 (8.6%) | 2 (1.7%) | 16 (13.8%) | 29 (25%) | 40 (34.5%) | 17 (14.7%) | 116 |
| 4. My experience in the school have helped me become knowledgeable about the health problems associated with various racial and cultural groups | 2 (1.7%) | 9 (7.8%) | 4 (3.4%) | 4 (3.4%) | 33 (28.4%) | 46 (39.7%) | 18 (15.5%) | 116 |
| 5. I think my beliefs and attitudes are influenced by my culture | 1 (0.9%) | 3 (2.6%) | 2 (1.7%) | 7 (6%) | 36 (31%) | 39 (33.6%) | 28 (24.1%) | 116 |
| 6. I think my behaviours are influenced by my culture | 2 (1.7%) | 0 (0%) | 1 (0.9%) | 10 (8.6%) | 35 (30.2%) | 45 (38.8%) | 23 (19.8%) | 116 |
| 7. I often reflect on how culture affects beliefs, attitudes and behaviours | 1 (0.9%) | 7 (6%) | 6 (5.2%) | 24 (20.7%) | 37 (31.9%) | 33 (28.4%) | 8 (6.9%) | 116 |
| 8. When I have an opportunity to help someone, I offer assistance less frequently to individuals of certain cultural backgrounds | 75 (64.7%) | 25 (21.6%) | 3 (2.6%) | 3 (2.6%) | 6 (5.2%) | 3 (2.6%) | 1 (0.9%) | 116 |
| 9. I am less patient with individuals of certain cultural backgrounds | 79 (68.1%) | 27 (23.3%) | 4 (3.4%) | 2 (1.7%) | 2 (1.7%) | 1 (0.9%) | 1 (0.9%) | 116 |
| 10. I feel comfortable working with patients from all ethnic groups | 0 (0%) | 0 (0%) | 2 (1.7%) | 2 (1.7%) | 3 (2.6%) | 32 (27.6%) | 77 (66.4%) | 116 |
| 11. I believe nurses own cultural beliefs influence their nursing care decisions | 1 (0.9%) | 4 (3.4%) | 2 (1.7%) | 17 (14.7%) | 42 (36.2%) | 34 (29.3%) | 16 (13.8%) | 116 |
| 12. I typically feel somewhat uncomfortable when I am in the company of people from cultural or ethnic backgrounds different from my own | 60 (51.7%) | 30 (25.9%) | 6 (5.2%) | 8 (6.9%) | 7 (6%) | 1 (0.9%) | 4 (3.4%) | 116 |
| 13. I have noticed that the nursing instructors call on students from minority cultural groups when issues related to them come up in class | 4 (3.4%) | 16 (13.8%) | 5 (4.3%) | 18 (15.5%) | 22 (19%) | 28 (24.1%) | 23 (19.8%) | 116 |
| 14. During group discussions or exercises, I have noticed the nursing instructors make an effort to ensure no student is excluded | 2 (1.7%) | 4 (3.4%) | 2 (1.7%) | 18 (15.5%) | 25 (21.6%) | 49 (42.2%) | 16 (13.8%) | 116 |
| 15. I think students’ cultural values influence their classroom behaviour (e.g. asking questions, participating in groups or offering comments) | 1 (0.9%) | 3 (2.6%) | 11 (9.6%) | 22 (19.1%) | 31 (27.0%) | 37 (32.2%) | 10 (8.7%) | 115 |
| 16. In my lectures and lab sessions my instructors have engaged in behaviours that may have made students from certain cultural backgrounds feel excluded | 20 (17.4%) | 41 (35.7%) | 13 (11.3%) | 26 (22.6%) | 10 (8.7%) | 5 (4.3%) | 0 (0%) | 115 |
| 17. I think it is the instructor’s responsibility to accommodate students’ diverse learning needs | 0 (0%) | 1 (0.9%) | 0 (0%) | 6 (5.2%) | 18 (15.5%) | 49 (42.2%) | 42 (36.2%) | 116 |
| 18. My nursing instructors seem comfortable discussing cultural issues in the classroom | 0 (0%) | 1 (0.9%) | 6 (5.2%) | 17 (14.7%) | 22 (19%) | 46 (39.7%) | 24 (20.7%) | 116 |
| 19. My nursing instructors seem interested in learning how their classroom behaviours may discourage students from certain cultural or ethnic groups | 1 (0.9%) | 8 (7%) | 9 (7.8%) | 41 (35.7%) | 21 (18.3%) | 31 (27%) | 4 (3.5%) | 115 |
| 20. I think the cultural values of the nursing instructors influence their behaviours in the classroom | 0 (0%) | 5 (4.3%) | 9 (7.8%) | 25 (21.7%) | 35 (30.4%) | 33 (28.7%) | 8 (7%) | 115 |
| 21. I believe the classroom experience in school helps students become more comfortable interacting with people from different cultures | 0 (0%) | 8 (6.9%) | 3 (2.6%) | 21 (18.1%) | 28 (24.1%) | 39 (33.6%) | 17 (14.7%) | 116 |
| 22. I believe some aspects of the classroom environment may alienate students from some cultural backgrounds | 6 (5.2%) | 28 (24.1%) | 10 (8.6%) | 32 (27.6%) | 26 (22.4%) | 9 (7.8%) | 5 (4.3%) | 116 |
| 23. I feel comfortable discussing cultural issues in the classroom | 0 (0%) | 6 (5.2%) | 17 (14.7%) | 14 (12.1%) | 33 (28.4%) | 35 (30.2%) | 11 (9.5%) | 116 |
| 24. My programme courses in the school have helped me become more comfortable interacting with people from different cultures | 1 (0.9%) | 5 (4.3%) | 3 (2.6%) | 22 (19%) | 22 (19%) | 37 (31.9%) | 26 (22.4%) | 116 |
| 25. I feel the nursing instructors respect differences in individuals from diverse cultural backgrounds | 0 (0%) | 0 (0%) | 7 (6%) | 7 (6%) | 22 (19%) | 60 (51.7%) | 20 (17.2%) | 116 |
| 26. The instructors model behaviours that are sensitive to multicultural issues | 2 (1.7%) | 10 (8.7%) | 5 (4.3%) | 34 (29.6%) | 23 (20%) | 34 (29.6%) | 7 (6.1%) | 115 |
| 27. The instructors use examples and or case studies that incorporate information from various cultures and ethnic groups | 3 (2.6%) | 5 (4.3%) | 9 (7.8%) | 9 (7.8%) | 23 (19.8%) | 56 (48.3%) | 11 (9.5%) | 116 |
| 28. I respect the decisions of my patients when they are influenced by their culture, even if I disagree | 0 (0%) | 1 (0.9%) | 0 (0%) | 3 (2.6%) | 10 (8.6%) | 46 (39.7%) | 56 (48.3%) | 116 |
| 29. If I need more information about a patient culture, I would use resources available onsite in school (e.g., books, audio or online resources) | 1 (0.9%) | 6 (5.2%) | 3 (2.6%) | 15 (12.9%) | 27 (23.3%) | 44 (37.9%) | 20 (17.2%) | 116 |
| 30. If I need more information about a patient culture, I would feel comfortable asking people I work with | 1 (0.9%) | 6 (5.2%) | 5 (4.3%) | 5 (4.3%) | 26 (22.4%) | 53 (45.7%) | 20 (17.2%) | 116 |
| 31. If I need more information about a patient culture, I would feel comfortable asking the patient or family member | 1 (0.9%) | 3 (2.6%) | 3 (2.6%) | 11 (9.5%) | 31 (26.7%) | 47 (40.5%) | 20 (17.2%) | 116 |
| 32. I feel somewhat uncomfortable working with the families of patients from cultural backgrounds different than my own | 57 (49.1%) | 29 (25%) | 7 (6%) | 8 (6.9%) | 7 (6%) | 5 (4.3%) | 3 (2.6%) | 116 |
