# Supplementary material for: Assessing cultural awareness and competency in nursing education: A cross-sectional study of undergraduate nursing students in Ireland
Source: PLoS One. 2026 Oct 12;21(10):e0359851. doi: 10.1371/journal.pone.0359851 (PMC13657305; doi:10.1371/journal.pone.0359851)
Supplement: Supplementary file 4 [file pone.0359851.s004.docx]

Dear Editor,

Thank you for your time in handling our manuscript. We have revised the manuscript in response to your comments and have highlighted the changes in the paper as requested. We have also provided detailed response to your comments. Please see our response below.

**Editor’s Comments**

1. The Subscale reliability is below acceptable thresholds. CA (α = 0.59) and BCI (α = 0.55) fall below the conventional 0.70 cutoff (some literature accepts a 0.60 threshold). The authors acknowledge this honestly and attribute it to short subscales, which is acceptable; however, this is not just a limitation to disclose; it means any non-significant finding on CA/BCI is uninterpretable (could be a true null or could be attenuation from unreliable measurement).

This point should be explicitly flagged in the Results or Discussion sections, particularly where the subscales are interpreted as if the null findings were essentially meaningful. For example, language such as “differences… not statistically significant” in the CA/BCI comparisons should be accompanied by acknowledgment that measurement unreliability may provide an alternative explanation for the null results.

Thank you for this comment. We have added a sentence to the Discussion section where we previously referred to non‑significant differences across the four subscale scores between students born in Ireland and those born outside Ireland, explicitly acknowledging the low reliability of the CA and BCI subscales.

“Given the lower reliability of the CA and BCI subscales, these non‑significant results should be interpreted cautiously, as limited measurement precision may obscure true differences.”

This has been added to the second paragraph of the Discussion and highlighted in green. As noted, this limitation was already addressed in the Strengths and Limitations section, so no further changes were required there. In the Conclusion, we only drew on statistically significant findings; therefore, no revisions were necessary in that section either.
