# Supplementary material for: Assessing cultural awareness and competency in nursing education: A cross-sectional study of undergraduate nursing students in Ireland
Source: PLoS One. 2026 Oct 12;21(10):e0359851. doi: 10.1371/journal.pone.0359851 (PMC13657305; doi:10.1371/journal.pone.0359851)
Supplement: Peer review history

# Peer review: Decision Letter 0

**DOI:** 10.1371/journal.pone.0359851.r001

**Authors:** Suad J Ghaben

17 Jun 2026

Dear Dr.  Hammoud,

Thank you for submitting your manuscript to PLOS ONE. After careful consideration, we feel that it has merit but does not fully meet PLOS ONE’s publication criteria as it currently stands. Therefore, we invite you to submit a revised version of the manuscript that addresses the points raised during the review process.

==============================

Editor's comments

- Please, clearly justify in the Methods section the use of purposive sampling.
- Please elaborate clearly in the Methods section, the exclusion of the Research Issues (RI) domain of the original 36-item CAS.
- Please confirm in the Methods whether the questionnaire used in this study (CAS-36 items excluding the RI) is validated, and if so, include the appropriate citation. If it is not validated, then please discuss whether the questionnaire was pilot-tested or included as one of the limitations in the Discussion. You should reflect on whether the exclusion of the RI section alters the psychometric properties of the CAS, especially the Construct Validity and the consistency of the scale (reliability). Identify if you calculated and reported a new alpha coefficient.
- Please discuss how the research findings could be generalized to other Irish nursing programs, given that the data were collected from a single institution.
- Please acknowledge in the study limitations the self-reporting bias and social desirability bias.
- Please ensure that your study reporting adheres to the relevant Equator network guideline:  https://www.equator-network.org/. Please do the following:

Please find the correct EQUATOR guideline for your study type, download the checklist, and fill it out. Please state in your Methods section that you have followed this guideline (name the specific guideline), provide a reference citation, and cite the completed checklist as a supplementary file. For example: ‘The reporting of this study conforms to the xxx statement (ref) [SUPPLEMENTAL FILE CITATION].

- Please consider redrafting the conclusion to reflect that it is supported by data rather than speculation or inference.
- Upon re-submission, authors should make sure they address each of the reviewers' and editors’ comments in a clear, point-by-point way; the changes/amendments must be clearly shown/marked in the resubmitted manuscript to facilitate a quick revision.

==============================

Please submit your revised manuscript by Aug 01 2026 11:59PM. If you will need more time than this to complete your revisions, please reply to this message or contact the journal office at plosone@plos.org. When you're ready to submit your revision, log on to https://www.editorialmanager.com/pone/ and select the 'Submissions Needing Revision' folder to locate your manuscript file.

- A letter that responds to each point raised by the academic editor and reviewer(s). You should upload this letter as a separate file labeled 'Response to Reviewers'.
- A marked-up copy of your manuscript that highlights changes made to the original version. You should upload this as a separate file labeled 'Revised Manuscript with Track Changes'.
- An unmarked version of your revised paper without tracked changes. You should upload this as a separate file labeled 'Manuscript'.

If you would like to make changes to your financial disclosure, please include your updated statement in your cover letter. Guidelines for resubmitting your figure files are available below the reviewer comments at the end of this letter.

If applicable, we recommend that you deposit your laboratory protocols in protocols.io to enhance the reproducibility of your results. Protocols.io assigns your protocol its own identifier (DOI) so that it can be cited independently in the future. For instructions see: https://journals.plos.org/plosone/s/submission-guidelines#loc-laboratory-protocols. Additionally, PLOS ONE offers an option for publishing peer-reviewed Lab Protocol articles, which describe protocols hosted on protocols.io. Read more information on sharing protocols at https://plos.org/protocols?utm_medium=editorial-email&utm_source=authorletters&utm_campaign=protocols.

As the corresponding author, your ORCID iD is verified in the submission system and will appear in the published article. PLOS supports the use of ORCID, and we encourage all coauthors to register for an ORCID iD and use it as well. Please encourage your coauthors to verify their ORCID iD within the submission system before final acceptance, as unverified ORCID iDs will not appear in the published article. *Only*  the individual author can complete the verification step; PLOS staff *cannot*  verify ORCID iDs on behalf of authors.

We look forward to receiving your revised manuscript.

Kind regards,

Suad J Ghaben, PhD

Academic Editor

PLOS One

Journal Requirements:

When submitting your revision, we need you to address these additional requirements.

1.Please ensure that your manuscript meets PLOS ONE's style requirements, including those for file naming. The PLOS ONE style templates can be found at

https://journals.plos.org/plosone/s/file?id=wjVg/PLOSOne_formatting_sample_main_body.pdf and

https://journals.plos.org/plosone/s/file?id=ba62/PLOSOne_formatting_sample_title_authors_affiliations.pdf

2.  If the reviewer comments include a recommendation to cite specific previously published works, please review and evaluate these publications to determine whether they are relevant and should be cited. There is no requirement to cite these works unless the editor has indicated otherwise.

Additional Editor Comments:

Thank you for your submission, the journal will revert back to you regarding further action.

[Note: HTML markup is below. Please do not edit.]

Reviewer's Responses to Questions

**Comments to the Author**

1. Is the manuscript technically sound, and do the data support the conclusions?

Reviewer #1: Yes

Reviewer #2: Partly

**********

2. Has the statistical analysis been performed appropriately and rigorously?

Reviewer #1: No

Reviewer #2: Yes

**********

3. Have the authors made all data underlying the findings in their manuscript fully available?

The PLOS Data policy

Reviewer #1: Yes

Reviewer #2: Yes

**********

4. Is the manuscript presented in an intelligible fashion and written in standard English?

Reviewer #1: Yes

Reviewer #2: Yes

**********

Reviewer #1: Dear Authors,

Thank you for submitting your manuscript, “Exploring Cultural Awareness and Competency in Nursing Education: A Demographic Analysis of Nursing Students in Ireland” . This is an important and timely topic, particularly as nursing education continues to respond to increasingly diverse healthcare environments. Your study addresses a meaningful gap and has clear potential to contribute to both educational practice and future research in this area.

The suggestions below are intended to support you in strengthening the clarity, rigor, and overall contribution of your manuscript.

Methodological clarity and transparency

The study design is appropriate and generally well described. However, several areas would benefit from further clarification. In particular, it would be helpful to provide a clearer rationale for the use of purposive sampling and to reflect on how this may affect the representativeness of the sample. Including a brief justification of sample size, even retrospectively, would also strengthen the study.

Given the response rate, a more explicit discussion of potential biases—such as selection bias, non-response bias, and social desirability bias—would add important context for interpreting the findings.

Measurement considerations

The use of the Cultural Awareness Scale is a strength of the study. However, as the instrument was modified, it is important to demonstrate how it performed in your sample. Reporting reliability statistics (such as Cronbach’s alpha) for the overall scale and subdomains would strengthen confidence in the results.

It would also be useful to more clearly explain the rationale for excluding the “Research Issues” domain and to reflect on how this decision may affect comparability with previous studies.

Statistical reporting

The analytical approach is appropriate, particularly the use of non-parametric tests. To enhance the interpretability of your findings, I encourage you to report effect sizes alongside p-values and, where possible, include confidence intervals.

You may also wish to consider additional analyses, such as regression models, to explore relationships between variables in a more comprehensive way.

Use and interpretation of demographic variables

The examination of demographic differences is a valuable aspect of the study. That said, the grouping of ethnicity into a binary variable would benefit from further consideration or clearer justification. A more nuanced discussion of these findings would help avoid oversimplification and better reflect the complexity of cultural identity.

Interpretation and discussion

The discussion is well connected to the literature and highlights relevant implications. To strengthen this section further, it would be helpful to ensure that interpretations remain consistent with the cross-sectional nature of the study. In particular, avoiding causal language and taking a more cautious approach to non-significant findings would improve the balance of the discussion.

Expanding the limitations section to more fully address measurement and analytical considerations would also strengthen the manuscript.

Presentation and minor points

A few small revisions would improve clarity and readability:

1. Consider including the study design in the title (e.g., “cross-sectional study”).

2. Address minor typographical and formatting inconsistencies.

3. Including a STROBE checklist as supplementary material would enhance reporting transparency.

Final remarks

This manuscript is grounded in an important area of inquiry and offers useful insights into cultural awareness in nursing education. With further attention to methodological detail, statistical reporting, and interpretation, the paper can be significantly strengthened.

I encourage you to revise the manuscript with these points in mind and look forward to seeing the next version of your work.

Reviewer #2: This manuscript addresses an important and timely topic by examining cultural awareness and competency among undergraduate nursing students in Ireland using the Cultural Awareness Scale (CAS). The paper is generally well structured, the study design is appropriate for the stated aims, and the topic aligns closely with ongoing efforts to advance diversity, equity, inclusion, and culturally competent care in nursing education. The authors are commended for conducting this study in an Irish context where demographic diversity is rapidly increasing and where evidence on nursing students' cultural awareness remains limited. The following comments are intended to help the authors strengthen the manuscript before publication.

Major Comments

1. Clarify the research gap and contribution

The background section provides a solid overview of policy and contextual drivers in the Irish healthcare and education systems, including relevant references to the National Intercultural Health Strategy, the NMBI curriculum review, and the growing diversity of Ireland's population and healthcare workforce. However, the specific research gap relative to previous CAS-based studies and other published work on cultural competence in undergraduate nursing is not fully articulated. Several studies have used the CAS tool in nursing education contexts across different countries, and the authors should clearly situate this study in relation to that body of evidence. It would considerably strengthen the paper to explain how this study advances existing literature, for example, whether it offers a new geographic or institutional context, a new demographic focus, or a new analytical approach, and to sharpen the main research questions so they are explicitly stated in the introduction and restated in the discussion. Without this, it is difficult for readers to assess the study's unique scientific contribution.

2. Study design limitation, sampling, representativeness, and generalisability

The manuscript does not explicitly acknowledge that a cross-sectional design cannot establish causality. This is important because certain passages in the discussion make causal-sounding claims, for example, the suggestion that "persons of colour perceive culturally inclusive practices less positively than White students" implies a directional relationship that the study design does not support. Cross-sectional data can only demonstrate associations at a single point in time; the authors should revise such statements accordingly and consistently frame their findings as associational rather than explanatory throughout the discussion and conclusions.

The use of purposive sampling from a single higher education institution, combined with a moderate response rate and a sample that is predominantly young (86.2% aged 18–24), female (93.1%), and White (72.4%), raises important questions about representativeness and generalisability. While the authors acknowledge some of these issues in the limitations section, the discussion of potential selection bias and its consequences for interpretation could be more explicit and systematic. Regarding the response rate specifically: a 48.5% participation rate (116 out of 239 eligible students) is moderate-to-low for a survey-based study. The authors should address whether there is any basis for inferring whether non-responders differ systematically from responders, for example, whether students who declined participation may have had lower interest in or exposure to diversity-related topics, which could introduce systematic bias in the reported CAS scores. If such information is not available, this should be explicitly acknowledged as a limitation that affects the interpretation of findings.

Furthermore, given that data were collected from a single institution, the authors should more explicitly discuss the extent to which findings can be generalised to other Irish nursing programmes, particularly those with different student demographic profiles or different approaches to integrating cultural competency content into their curricula. While the authors note that the institution is one of the largest in Ireland, this alone does not ensure representativeness.

3. Self-report bias and social desirability

The CAS is an entirely self-reported instrument, and the study does not include any triangulation through alternative data sources such as faculty observation, behavioural assessments, or clinical placement evaluations. The authors should explicitly acknowledge self-report bias and, in particular, social desirability bias as a potential limitation. This is especially relevant in the context of a culturally sensitive topic such as cultural awareness and competency, where participants may respond in ways they perceive to be socially acceptable or professionally expected rather than reflecting their actual attitudes, knowledge, or behaviours. The limitations section should include a clear statement acknowledging this risk and its potential impact on the validity of the findings, particularly the statistically significant differences observed between White participants and participants who identified as people of colour.

4. Handling of ethnicity and categorisation

The decision to collapse multiple ethnic categories into a binary variable comprising "White" and "People of Colour" is understandable given the small subgroup sizes in the sample (32 participants identified as people of colour). However, this approach combines potentially very heterogeneous populations, including individuals of Black, Asian, Arab, and other backgrounds into a single analytical category, which may obscure important between-group differences in cultural awareness, experiences of education, and perceptions of inclusive practice. The authors are encouraged to provide a stronger methodological justification for this decision in the methods section, including a description of the original underlying categories before they were collapsed and a clear statement of the analytical rationale. When interpreting the statistically significant differences between these two broad groups in the discussion, more caution is warranted. The authors should explicitly acknowledge that the dichotomy may mask important nuances and that findings for "people of colour" as a group should not be generalised across the diverse subpopulations it encompasses. The discussion should also reflect more carefully on whether the observed differences in GEE and Total Awareness scores between White participants and people of colour may reflect differences in educational experience, institutional belonging, or responses to bias rather than differences in cultural awareness per se.

5. Confounding variables, statistical analysis, and temporal validity

The analytical approach examines CAS scores across demographic variables independently using non-parametric tests. While this is appropriate for bivariate comparisons, it does not account for the possibility that observed associations between demographic characteristics and cultural awareness scores may be confounded by other variables. For example, year of study, volume of clinical placement hours, prior international experience, language background, or socioeconomic status may independently influence CAS scores and may also be correlated with the demographic variables examined. The authors should consider whether a multivariate analysis, such as multiple regression or ordinal regression, would be feasible given the sample size, and if so, report such results or at minimum discuss this as a limitation and direction for future research. If multivariate analysis is not feasible given the current sample size, a post-hoc power analysis for the key subgroup comparisons (particularly the ethnicity comparison with n=32 in the people of colour group) would help readers assess the robustness of the findings.

Additionally, data were collected in November 2022 and January 2023. The authors should reflect on whether findings remain applicable given Ireland's rapidly changing demographic landscape since that period, and whether the timing of data collection at the end of the first semester and the beginning of the second semester respectively may have influenced scores, particularly for subscales related to educational experience (GEE), which might vary depending on how much curriculum content students had been exposed to at the time of data collection.

6. Interpretation of findings and strength of conclusions

The manuscript at times moves quickly from descriptive findings to relatively broad conclusions about institutional practices and student experiences. To enhance scientific rigour and credibility, the authors should more clearly and consistently distinguish between conclusions that are directly and strongly supported by the data and interpretations that are more speculative or inferential. For example, the suggestion that persons of colour perceive culturally inclusive practices in education less positively than White students is a plausible and important finding; however, the study design does not permit exploration of the underlying mechanisms, and the authors should be explicit about this boundary. Where interpretations go beyond the data, the authors should use appropriately cautious language (e.g., "these findings may suggest," "one possible interpretation is") and should explicitly call for qualitative or mixed-methods follow-up studies to explore mechanisms and lived experiences in greater depth.

7. Breadth of implications and alignment with evidence

The implications for practice and education presented in the discussion are extensive and wide-ranging, but some are only loosely connected to the evidence generated by this study. The discussion introduces a variety of strategies, including healthcare simulation, patient and public involvement, universal design for learning, leadership development through critical consciousness, and co-design of curricula many of which are not directly examined or supported by the study's own data. While reference to broader literature is appropriate and valuable, the authors should more explicitly distinguish between recommendations that flow directly from their own findings and those that are drawn from the wider literature. A useful approach would be to begin the implications section with what the study's own findings specifically suggest, and then broaden to evidence-informed recommendations from the literature as complementary rather than primary evidence. This would ensure that the study's empirical contribution is not overstated and that readers can accurately assess the evidentiary basis for each recommendation.

Minor Comments

1. Measurement properties and reporting

Since the CAS is the central instrument of this study, it would considerably strengthen the manuscript to report internal consistency indices, specifically Cronbach's alpha for each of the four subscales and the total scale as measured in this sample, even if reliability has been reported in previous studies using the CAS. Reliability can vary across samples and contexts, and reporting these values will reassure readers about the appropriateness and stability of the instrument in the Irish undergraduate nursing context. If alpha values were calculated and are satisfactory, this should be reported; if they were not calculated, the authors should consider doing so.

2. Statistical reporting and table clarity

The use of non-parametric tests (Mann-Whitney U and Kruskal-Wallis) is appropriate given the non-normal distribution of the data as confirmed by the Shapiro-Wilk test. However, the presentation of results in the tables could be further standardised to improve readability and professionalism. Specifically, the authors should ensure consistent use of decimal places for mean ranks and p-values across all tables, consider bolding or otherwise visually highlighting statistically significant results to aid rapid interpretation, and include clear table legends and footnote explanations for all abbreviations and statistical tests used. These are standard expectations for PLOS ONE publications and will improve the clarity of the results section.

3. Terminology and consistency

Several key concepts, including cultural awareness, cultural competence, person-centred care, and people of colour appear throughout the manuscript but are not always defined consistently or used with the same meaning. The authors should define each of these terms clearly and precisely on first use and then apply them consistently throughout the manuscript. This is particularly important for an international readership, where these terms may carry different connotations across healthcare, education, and research contexts.

4. Excluded domain

The Research Issues (RI) domain of the original 36-item CAS was excluded from this study on the basis that undergraduate nursing students have limited research experience. While this rationale is understandable, it is not elaborated upon in sufficient methodological detail. The authors should clarify whether the exclusion of this domain affects comparability of their total CAS scores with those reported in previous studies that used the full 36-item version, and should discuss whether any re-validation or adaptation of the modified 32-item version was undertaken or whether the existing psychometric properties of the full scale can reasonably be assumed to apply to the modified version used here.

5. Language and style

Overall, the manuscript is clearly written and accessible. A careful proofread or light language editing pass to address minor typographical errors, grammatical inconsistencies, and formatting irregularities would enhance the overall professionalism and readability of the manuscript. This does not require major rewriting, but attention to detail at this level is expected for publication in a peer-reviewed journal.

Overall Assessment

Overall, this manuscript addresses an important and timely topic, providing valuable insight into cultural awareness among undergraduate nursing students in Ireland and highlighting meaningful equity and inclusion issues within nursing education. The study is generally well structured, uses an appropriate and validated instrument, and produces findings that are broadly consistent with the existing international literature. However, several methodological concerns require substantive attention before the manuscript can be recommended for publication. These include the need to acknowledge the cross-sectional design's inability to establish causality, address potential self-report and social desirability bias, provide a more thorough discussion of non-response bias, justify the exclusion of the RI domain and its implications for comparability, consider multivariate analysis to account for confounding variables, and ensure that all interpretive claims are appropriately hedged and aligned with the study's empirical evidence. The treatment of ethnicity categorisation also requires stronger methodological justification. With these revisions, the paper has good potential to make a meaningful contribution to the literature on cultural competence and nursing education, and to inform curriculum development and institutional policy in the Irish and broader international nursing education context.

**********

what does this mean?). If published, this will include your full peer review and any attached files.

If you choose “no”, your identity will remain anonymous but your review may still be made public.

**Do you want your identity to be public for this peer review?**  For information about this choice, including consent withdrawal, please see our Privacy Policy

Reviewer #1: No

Reviewer #2: **Yes:**  Mazen Abuqamar

**********

[NOTE: If reviewer comments were submitted as an attachment file, they will be attached to this email and accessible via the submission site. Please log into your account, locate the manuscript record, and check for the action link "View Attachments". If this link does not appear, there are no attachment files.]

To ensure your figures meet our technical requirements, please review our figure guidelines: https://journals.plos.org/plosone/s/figures

You may also use PLOS’s free figure tool, NAAS, to help you prepare publication quality figures: https://journals.plos.org/plosone/s/figures#loc-tools-for-figure-preparation.

NAAS will assess whether your figures meet our technical requirements by comparing each figure against our figure specifications.

# Author comment: Author response to Decision Letter 1

**DOI:** 10.1371/journal.pone.0359851.r002

22 Jul 2026

Dear Editor,

Thank you for your time in handling our manuscript. We have revised the manuscript in response to the reviewers’ comments and have highlighted the changes in the paper as requested. We have also provided detailed responses to the reviewers’ comments. PPlease see our responses below. In addition, we have prepared a point‑by‑point table to make it easier for you to follow our replies. We have uploaded this document titled “Responses to reviewers.”

Editor

1. Please, clearly justify in the Methods section the use of purposive sampling.

Authors response: This has now been addressed. Please see our response to Reviewer 1, Comment 1.

2. Please elaborate clearly in the Methods section, the exclusion of the Research Issues (RI) domain of the original 36-item CAS.

Authors response: This has now been addressed. Please see our response to Reviewer 1, Comment 5, and Reviewer 2, Comment 15.

3. Please confirm in the Methods whether the questionnaire used in this study (CAS-36 items excluding the RI) is validated, and if so, include the appropriate citation. If it is not validated, then please discuss whether the questionnaire was pilot-tested or included as one of the limitations in the Discussion. You should reflect on whether the exclusion of the RI section alters the psychometric properties of the CAS, especially the Construct Validity and the consistency of the scale (reliability). Identify if you calculated and reported a new alpha coefficient.

Authors response: Thank you for this important comment. We have expanded the description of the instrument in the Methods section, including a detailed explanation of the face validity evaluation conducted by five expert reviewers. We have also calculated and reported Cronbach’s alpha coefficients for the total scale and each of the four subscales, and we comment on these values in relation to findings from existing studies (see Instrument and Variables subsection, page 8).

Additionally, we have added a limitation acknowledging that the removal of the RI domain may have influenced the psychometric properties of the modified CAS, particularly construct validity, as a full validation was not feasible within the scope of this study (see Limitations subsection, page 18).

4. Please discuss how the research findings could be generalized to other Irish nursing programs, given that the data were collected from a single institution.

Authors response: This has now been addressed. Please see our response to Reviewer 2, Comment 4.

5. Please acknowledge in the study limitations the self-reporting bias and social desirability bias.

Authors response: This has now been addressed. Please see our responses to Reviewer 1, Comment 3, and Reviewer 2, Comments 3 and 5.

6. Please ensure that your study reporting adheres to the relevant Equator network guideline: https://www.equator-network.org/. Please do the following: Please find the correct EQUATOR guideline for your study type, download the checklist, and fill it out. Please state in your Methods section that you have followed this guideline (name the specific guideline), provide a reference citation, and cite the completed checklist as a supplementary file.

Authors response: The full STROBE checklist has now been provided as a supplementary file (S1 Appendix), and it is explicitly cited in the Methods section under the Study Design and Setting subsection.

7. Please consider redrafting the conclusion to reflect that it is supported by data rather than speculation or inference.

Authors response: The conclusion has been redrafted to reflect data rather than speculation.

8. Upon re-submission, authors should make sure they address each of the reviewers' and editors’ comments in a clear, point-by-point way; the changes/amendments must be clearly shown/marked in the resubmitted manuscript to facilitate a quick revision.

Authors response: We have responded to and addressed all comments from both reviewers and the editor in this point-by-point table. All changes and additions to the manuscript have been highlighted in blue font to facilitate a quick review.

Reviewer One

Thank you for submitting your manuscript, “Exploring Cultural Awareness and Competency in Nursing Education: A Demographic Analysis of Nursing Students in Ireland”. This is an important and timely topic, particularly as nursing education continues to respond to increasingly diverse healthcare environments. Your study addresses a meaningful gap and has clear potential to contribute to both educational practice and future research in this area.

The suggestions below are intended to support you in strengthening the clarity, rigor, and overall contribution of your manuscript.

Authors response: Thank you very much for your feedback and for taking the time to review our manuscript.

Methodological clarity and transparency

1. The study design is appropriate and generally well described. However, several areas would benefit from further clarification. In particular, it would be helpful to provide a clearer rationale for the use of purposive sampling and to reflect on how this may affect the representativeness of the sample.

Authors response: Thank you for this helpful comment. We have expanded the rationale for using purposive sampling as follows:

Purposive sampling was used because the study aimed to assess cultural awareness among nursing students who had sufficient academic and clinical exposure to meaningfully reflect on cultural encounters. Students in years three and four have completed substantial theoretical modules and multiple clinical placements, providing the experiential foundation required to engage with the subscales measured by the CAS. Year one and year two students were excluded because they have limited clinical exposure and may not yet have encountered culturally diverse situations in practice, making their inclusion less aligned with the study aim.

We have also added a reflection on how this sampling approach may affect representativeness in the limitations subsection (page 19).

2. Including a brief justification of sample size, even retrospectively, would also strengthen the study.

Authors response: Thank you for this comment. We have now added a brief justification of the sample size in the Methods section. As this study aimed to survey all eligible undergraduate nursing students in years 3 and 4, a formal sample size calculation was not conducted. Instead, all students who met the inclusion criteria (N = 239) were invited to participate, and the final sample of 116 respondents represents the maximum achievable sample from this defined population. This clarification has been added to the Study sample and eligibility criteria subsection on page 7.

3. Given the response rate, a more explicit discussion of potential biases—such as selection bias, non-response bias, and social desirability bias—would add important context for interpreting the findings.

Authors response: Thank you for this important comment. This issue was raised by both reviewers, and we have now addressed it more explicitly in the limitations section. We discuss the moderate-to-low response rate, and the potential for selection bias, non‑response bias, and social desirability bias, and outline how these may have influenced the findings and their generalisability. Please see the revised limitations subsection (page 19).

Measurement considerations

4. The use of the Cultural Awareness Scale is a strength of the study. However, as the instrument was modified, it is important to demonstrate how it performed in your sample. Reporting reliability statistics (such as Cronbach’s alpha) for the overall scale and subdomains would strengthen confidence in the results.

Authors response: Thank you for this important comment. We have expanded the description of the instrument in the Methods section, including a detailed explanation of the face validity evaluation conducted by five expert reviewers. We have also calculated and reported Cronbach’s alpha coefficients for the total scale and each of the four subscales, and we comment on these values in relation to findings from existing studies (see Instrument and Variables subsection, page 8).

Additionally, we have added a limitation acknowledging that the removal of the RI domain may have influenced the psychometric properties of the modified CAS, particularly construct validity, as a full validation was not feasible within the scope of this study (see Limitations subsection, page 18).

5. It would also be useful to more clearly explain the rationale for excluding the “Research Issues” domain and to reflect on how this decision may affect comparability with previous studies.

Authors response: Thank you for this comment. We have now elaborated clearly in the Methods section on the exclusion of the RI domain. Specifically, we explain that the RI items focus on research engagement and decision‑making processes that are not applicable to undergraduate nursing students, who typically have limited research experience and at this stage of their programme, and have not yet completed thesis or dissertation work. This decision was also reviewed as part of the face‑validity assessment conducted by five expert reviewers (three senior nursing lecturers and two clinical tutors), who confirmed that the RI domain was not relevant for this population. As a result, the RI domain was removed, and the final instrument used in this study comprised 32 items. This rationale is now detailed in the Instrument and Variables subsection (page 8).

We also acknowledge that this modification has implications for comparability with previous studies using the full 36 item CAS. Specifically, the exclusion of the RI domain limits comparability with studies reporting total CAS scores based on all five original domains; however, comparability across the four retained subscales remains intact, as no items within these domains were removed or modified. This clarification has been added to the Limitations section (page 19).

Statistical reporting

6. The analytical approach is appropriate, particularly the use of non-parametric tests. To enhance the interpretability of your findings, I encourage you to report effect sizes alongside p-values and, where possible, include confidence intervals.

Authors response: Thank you for this suggestion. We agree that reporting effect sizes enhances the interpretability of non‑parametric results. We have now added effect sizes (r for Mann-Whitney U and η² for Kruskal-Wallis) to the relevant table. As confidence intervals for mean ranks are not typically reported in non‑parametric analyses, we did not calculate them. We therefore report effect sizes, which are the recommended indicators of magnitude for Mann–Whitney U and Kruskal–Wallis tests. Please see Table 3 on page 14, Cultural awareness scores across demographics subsection on page 13, and the discussion section on page 15.

7. You may also wish to consider additional analyses, such as regression models, to explore relationships between variables in a more comprehensive way.

Authors response: Thank you for this suggestion. We considered multivariate analysis; however, due to small subgroup sizes and the risk of unstable parameter estimates, regression modelling was not feasible. Although the observed effect sizes were small‑to‑moderate, effects of this magnitude typically require substantially larger samples to support adequately powered and stable multivariate models.

We have clarified this in the Methods (Data analysis subsection) and added a statement in the Discussion and Limitations recommending multivariate approaches for future research.

Use and interpretation of demographic variables

8. The examination of demographic differences is a valuable aspect of the study. That said, the grouping of ethnicity into a binary variable would benefit from further consideration or clearer justification. A more nuanced discussion of these findings would help avoid oversimplification and better reflect the complexity of cultural identity.

Authors response: Thank you for this important comment. We agree that cultural identity is complex and that collapsing ethnicity into a binary variable risks oversimplifying this diversity. We have now added a clearer justification in the Methods section (data analysis subsection, page 10) explaining the original categories and the statistical constraints that led to this decision. We also expanded the Discussion to acknowledge the heterogeneity within the “people of colour” group and the implications for interpreting these findings (please see page 16-17)

Interpretation and discussion

9. The discussion is well connected to the literature and highlights relevant implications. To strengthen this section further, it would be helpful to ensure that interpretations remain consistent with the cross-sectional nature of the study. In particular, avoiding causal language and taking a more cautious approach to non-significant findings would improve the balance of the discussion.

Authors response: Thank you for this helpful comment. We have revised the Discussion to ensure that all interpretations are consistent with the cross‑sectional nature of the study. We removed causal language, framed all findings as associations, and adopted a more cautious approach when discussing non‑significant results.

10. Expanding the limitations section to more fully address measurement and analytical considerations would also strengthen the manuscript.

Authors response: We have expanded the Limitations section to more fully address both measurement and analytical considerations. Specifically, we have added:

purposive sampling and its implications for representativeness, the moderate‑to‑low response rate and potential non‑response bias, the self‑report and social desirability bias associated with the CAS instrument, the removal of the RI domain, the dichotomous grouping of ethnicity, reliance on non‑parametric bivariate analyses due to the non‑feasibility of multivariate modelling, and the limited statistical power associated with the small number of participants categorised as people of colour (n = 32).

Presentation and minor points

A few small revisions would improve clarity and readability:

11. Consider including the study design in the title (e.g., “cross-sectional study”).

Authors response: We have revised the title to include the study design. The updated title is: Assessing Cultural Awareness and Competency in Nursing Education: A Cross-sectional Study of Undergraduate Nursing Students in Ireland.

12. Address minor typographical and formatting inconsistencies.

Authors response: The manuscript has been revised for typographical and formatting inconsistencies.

13. Including a STROBE checklist as supplementary material would enhance reporting transparency.

Authors response: The full STROBE checklist has now been provided as a supplementary file (S1 Appendix), and it is explicitly cited in the Methods section under the Study Design and Setting subsection.

Final remarks

This manuscript is grounded in an important area of inquiry and offers useful insights into cultural awareness in nursing education. With further attention to methodological detail, statistical reporting, and interpretation, the paper can be significantly strengthened.

I encourage you to revise the manuscript with these points in mind and look forward to seeing the next version of your work.

Authors response: Thank you very much for your feedback.

Reviewer Two

This manuscript addresses an important and timely topic by examining cultural awareness and competency among undergraduate nursing students in Ireland using the Cultural Awareness Scale (CAS). The paper is generally well structured, the study design is appropriate for the stated aims, and the topic aligns closely with ongoing efforts to advance diversity, equity, inclusion, and culturally competent care in nursing education. The authors are commended for conducting this study in an Irish context where demographic diversity is rapidly increasing and where evidence on nursing students' cultural awareness remains limited. The following comments are intended to help the authors strengthen the manuscript before publication.

Authors response: Thank you very much for your feedback and for taking the time to review our manuscript.

Major Comments

Clarify the research gap and contribution

1. The background section provides a solid overview of policy and contextual drivers in the Irish healthcare and education systems, including relevant references to the National Intercultural Health Strategy, the NMBI curriculum review, and the growing diversity of Ireland's population and healthcare workforce. However, the specific research gap relative to previous CAS-based studies and other published work on cultural competence in undergraduate nursing is not fully articulated. Several studies have used the CAS tool in nursing education contexts across different countries, and the authors should clearly situate this study in relation to that body of evidence. It would considerably strengthen the paper to explain how this study advances existing literature, for example, whether it offers a new geographic or institutional context, a new demographic focus, or a new analytical approach, and to sharpen the main research questions so they are explicitly stated in the introduction and restated in the discussion. Without this, it is difficult for readers to assess the study's unique scientific contribution.

Authors response: Thank you for this important comment. We have strengthened the Background section by more clearly articulating the research gap in relation to previous CAS‑based studies in nursing education. Specifically, we now situate this study within the existing international literature and highlight how it contributes new evidence from an Irish undergraduate nursing context, where CAS has not previously been applied. We also emphasise the demographic focus of the study and the analytical approach used to examine socio‑demographic variation in cultural awareness.

In addition, we have refined and aligned the study aim across the Abstract, Introduction, and Discussion to ensure consistency and to clearly state the focus of the study.

Study design limitation, sampling, representativeness, and generalisability

2. The manuscript does not explicitly acknowledge that a cross-sectional design cannot establish causality. This is important because certain passages in the discussion make causal-sounding claims, for example, the suggestion that "persons of colour perceive culturally inclusive practices less positively than White students" implies a directional relationship that the study design does not support. Cross-sectional data can only demonstrate associations at a single point in time; the authors should revise such statements accordingly and consistently frame their findings as associational rather than explanatory throughout the discussion and conclusions.

Authors response: Thank you for highlighting this important point. We have revised the Discussion and Conclusion to ensure that all statements reflect associations rather than directional or explanatory claims. Phrases that previously implied causality have been reworded to emphasise patterns of association observed at a single point in time.

3. The use of purposive sampling from a single higher education institution, combined with a moderate response rate and a sample that is predominantly young (86.2% aged 18–24), female (93.1%), and White (72.4%), raises important questions about representativeness and generalisability. While the authors acknowledge some of these issues in the limitations section, the discussion of potential selection bias and its consequences for interpretation could be more explicit and systematic. Regarding the response rate specifically: a 48.5% participation rate (116 out of 239 eligible students) is moderate-to-low for a survey-based study. The authors should address whether there is any basis for inferring whether non-responders differ systematically from responders, for example, whether students who declined participation may have had lower interest in or exposure to diversity-related topics, which could introduce systematic bias in the reported CAS scores. If such information is not available, this should be explicitly acknowledged as a limitation that affects the interpretation of findings.

Authors response: Thank you for this important comment. We agree that representativeness and generalisability require more explicit discussion. We have now strengthened the limitations section to more clearly acknowledge that the sample was predominantly young, female, and White which reflects the demographic profile of nursing programmes in Ireland (Central Statistical Office, 2022), but nonetheless may limits representativeness, and that this demographic profile may limit the generalisability of the findings to more diverse nursing student populations.

We also added a discussion on non response bias. The response rate was moderate to low, and we recognise that students who chose not to participate may differ systematically from responders, for example in their interest in or exposure to diversity related topics. As no data were collected on non responders, we cannot determine whether such differences exist or how they may have influenced the CAS scores. This has now been explicitly acknowledged in the limitations subsection (page 19).

4. Furthermore, given that data were collected from a single institution, the authors should more explicitly discuss the extent to which findings can be generalised to other Irish nursing programmes, particularly those with different student demographic profiles or different approaches to integrating cultural competency content into their curricula. While the authors note that the institution is one of the largest in Ireland, this alone does not ensure representativeness.

Authors response: We agree that collecting data from a single institution limits generalisability. We have revised the Limitations section to more explicitly acknowledge that, although the institution is one of the largest in Ireland, this may not ensure representativeness of all Irish nursing programmes. We now note that demographic profiles and approaches to integrating cultural competency content may differ across institutions, and we recommend multi‑site studies in future research to strengthen generalisability.

Self-report bias and social desirability

5. The CAS is an entirely self-reported instrument, and the study does not include any triangulation through alternative data sources such as faculty observation, behavioural assessments, or clinical placement evaluations. The authors should explicitly acknowledge self-report bias and, in particular, social desirability bias as a potential limitation. This is especially relevant in the context of a culturally sensitive topic such as cultural awareness and competency, where participants may respond in ways they perceive to be socially acceptable or professionally expected rather than reflecting their actual attitudes, knowledge, or behaviours. The limitations section should include a clear statement acknowledging this risk and its potential impact on the validity of the findings, particularly the statistically significant differences observed between White participants and participants who identified as people of colour.

Authors response: Thank you for this important comment. We agree that the self-reported nature of the CAS introduces the possibility of self-report bias and, in particular, social desirability bias. We have now acknowledged this in the limitation subsection.

Given the culturally sensitive nature of cultural awareness and competency, participants may have responded in ways they perceived to be socially acceptable or professionally expected. We also note that this bias may have influenced the statistically significant differences observed between White participants and participants who were categorised as people of colour on the GEE subscale and the total CAS score.

A clear statement addressing this potential bias and its implications for interpreting the findings has been added to the limitations subsection (page 19).

Handling of ethnicity and categorisation

6. The decision to collapse multiple ethnic categories into a binary variable comprising "White" and "People of Colour" is understandable given the small subgroup sizes in the sample (32 participants identified as people of colour). However, this approach combines potentially very heterogeneous populations, including individuals of Black, Asian, Arab, and other backgrounds into a single analytical category, which may obscure important between-group differences in cultural awareness, experiences of education, and perceptions of inclusive practice. The authors are encouraged to provide a stronger methodological justification for this decision in the methods section, including a description of the original underlying categories before they were collapsed and a clear statement of the analytical rationale.

Authors response: Thank you for this important point. We have now provided a stronger methodological justification for collapsing the six original ethnicity categories into a binary variable, including a description of the original categories and the small subgroup sizes that limited meaningful statistical comparison (data analysis subsection, page 11). We also added a statement in the Discussion acknowledging that this approach combines heterogeneous populations and may obscure between group differences. Future research with larger samples is recommended to explore these differences in more detail (please see pages 16-17).

7. When interpreting the statistically significant differences between these two broad groups in the discussion, more caution is warranted. The authors should explicitly acknowledge that the dichotomy may mask important nuances and that findings for "people of colour" as a group should not be generalised across the diverse subpopulations it encompasses. The discussion should also reflect more carefully on whether the observed differences in GEE and Total Awareness scores between White participants and people of colour may reflect differences in educational experience, institutional belonging, or responses to bias rather than differences in cultural awareness per se.

Authors response: Thank you for this important comment. We agree that the binary ethnicity variable may mask important subgroup differences and that quantitative scores alone cannot fully capture the complexity of students’ cultural experiences. We have revised the Discussion to explicitly acknowledge this limitation and to caution against generalising findings across diverse subpopulations. We also added a recommendation for future research using larger samples and mixed‑methods or qualitative approaches to explore these nuances more deeply, as suggested (Please see pages 16-17).

Confounding variables, statistical analysis, and temporal validity

8. The analytical approach examines CAS scores across demographic variables independently using non-parametric tests. While this is appropriate for bivariate comparisons, it does not account for the possibility that observed associations between demographic characteristics and cultural awareness scores may be confounded by other variables. For example, year of study, volume of clinical placement hours, prior international experience, language background, or socioeconomic status may independently influence CAS scores and may also be correlated with the demographic variables examined. The authors should consider whether a multivariate analysis, such as multiple regression or ordinal regression, would be feasible given the sample size, and if so, report such results or at minimum discuss this as a limitation and direction for future research. If multivariate analysis is not feasible given the current sample size, a post-hoc power analysis for the key subgroup comparisons (particularly the ethnicity comparison with n=32 in the people of colour group) would help readers assess the robustness of the findings.

Authors response: Thank you for this important comment. We agree that several additional variables, such as placement hours, international experience, language background, and socioeconomic status, may influence cultural awareness and perceptions of inclusivity. These variables were not collected in the present study, as the focus was on demographic characteristics. We have now acknowledged this in the Limitations and recommended that future research incorporate these variables to enable a more comprehensive analysis.

We have also added a methodological justification (data analysis subsection) explaining why multivariate analysis was not feasible given the overall sample size and small subgroup counts, particularly the people of colour group (n = 32). The Discussion and Limitations sections have been expanded to note the possibility of confounding and to highlight that the small subgroup size may limit statistical power. Finally, we have included a recommendation for future research using larger samples and multivariate approaches to explore these relationships in greater depth.

9. Additionally, data were collected in November 2022 and January 2023. The authors should reflect on whether findings remain applicable given Ireland's rapidly changing demographic landscape since that period, and whether the timing of data collection at the end of the first semester and the beginning of the second semester respectively may have influenced scores, particularly for subscales related to educational experience (GEE), which might vary depending on how much curriculum content students had been exposed to at the time of data collection.

Authors response: Thank you for this comment. We have expanded the Limitations section to address both issues raised. First, we now acknowledge that data were collected in late 2022 and early 2023, and that Ireland’s demographic landscape has continued to evolve since that period, which may affect the applicability of the findings. Second, we note that the timing of data collection - at the end of the first semester and the beginning of the second - may have influenced scores on subscales related to educational experience, particularly GEE, due to varying levels of curricular exposure at different points in the academic year. We have incorporated both points into the revised manuscript and recommend future studies that collect data across multiple time points to better capture temporal variation.

Interpretation of findings and strength of conclusions

10. The manuscript at times moves quickly from descriptive findings to relatively broad conclusions about institutional practices and student experiences. To enhance scientific rigour and credibility, the authors should more clearly and consistently distinguish between conclusions that are directly and strongly supported by the data and interpretations that are more speculative or inferential. For example, the suggestion that persons of colour perceive culturally inclusive practices in education less positively than White students is a plausible and important finding; however, the study design does not permit exploration of the underlying mechanisms, and the authors should be explicit about this boundary. Where interpretations go beyond the data, the authors should use appropriately cautious language (e.g., "these findings may suggest," "one possible interpretation is") and should explicitly call for qualitative or mixed-methods follow-up studies to explore mechanisms and lived experiences in greater depth.

Authors response: Thank you for this helpful comment. We have revised the Discussion to more clearly distinguish between findings directly supported by the data and interpretations that are more inferential. We have strengthened the wording to reflect the associative nature of the study design, adopted more cautious language where interpretations extend beyond the data, and added a call for future qualitative or mixed methods research to provide richer insights into the underlying mechanisms.

Breadth of implications and alignment with evidence

11. The implications for practice and education presented in the discussion are extensive and wide-ranging, but some are only loosely connected to the evidence generated by this study. The discussion introduces a variety of strategies, including healthcare simulation, patient and public involvement, universal design for learning, leadership development through critical consciousness, and co-design of curricula many of which are not directly examined or supported by the study's own data. While reference to broader literature is appropriate and valuable, the authors should more explicitly distinguish between recommendations that flow directly from their own findings and those that are drawn from the wider literature. A useful approach would be to begin the implications section with what the study's own findings specifically suggest, and then broaden to evidence-informed recommendations from the literature as complementary rather than primary evidence. This would ensure that the study's empirical contribution is not overstated and that readers can accurately assess the evidentiary basis for each recommendation.

Authors response: Thank you for this important comment. We fully agree that the implications for practice and education must be clearly distinguished from the findings generated by the study and should not overstate the empirical contribution of a cross‑sectional design.

In response, we substantially revised the Discussion and Implications sections to ensure that recommendations flowing directly from our findings are clearly separated from broader evidence‑informed strategies drawn from the literature.

The previously mentioned strategies (co-design, leadership, PPI, etc..) are now clearly framed as approaches supported by wider literature, rather than as recommendations arising from our findings.

-We restructured the Implications section so that it now begins with the study’s own findings and presents only those implications that are directly supported by the data.

-We revised the Conclusion to ensure it reflects only the study’s empirical findings and appropriate associative interpretations.

Minor Comments

Measurement properties and reporting

12. Since the CAS is the central instrument of this study, it would considerably strengthen the manuscript to report internal consistency indices, specifically Cronbach's alpha for each of the four subscales and the total scale as measured in this sample, even if reliability has been reported in previous studies using the CAS. Reliability can vary across samples and contexts, and reporting these values will reassure readers about the appropriateness and stability of the instrument in the Irish undergraduate nursing context. If alpha values were calculated and are satisfactory, this should be reported; if they were not calculated, the authors should consider doing so.

Authors response: Thank you for this important comment. This point was also raised by the editor and Reviewer 1. We have expanded the description of the instrument in the Methods section, including a detailed explanation of the face validity evaluation conducted by five expert reviewers. We have also calculated and reported Cronbach’s alpha coefficients for the total scale and each of the four subscales, and we comment on these values in relation to findings from existing studies (see Instrument and Variables subsection, page 8).

Additionally, we have added a limitation acknowledging that the removal of the RI domain may have influenced the psychometric properties of the modified CAS, particularly construct validity, as a full validation was not feasible within the scope of this study (see Limitations subsection, page 19).

Statistical reporting and table clarity

13. The use of non-parametric tests (Mann-Whitney U and Kruskal-Wallis) is appropriate given the non-normal distribution of the data as confirmed by the Shapiro-Wilk test. However, the presentation of results in the tables could be further standardised to improve readability and professionalism. Specifically, the authors should ensure consistent use of decimal places for mean ranks and p-values across all tables, consider bolding or otherwise visually highlighting statistically significant results to aid rapid interpretation, and include clear table legends and footnote explanations for all abbreviations and statistical tests used. These are standard expectations for PLOS ONE publications and will improve the clarity of the results section.

Authors response: Thank you for this helpful suggestion. We have now standardised the presentation of all tables to improve clarity and alignment with PLOS ONE formatting expectations. Mean ranks are consistently reported to two decimal places, p‑values to three decimal places, and effect sizes (r for Mann-Whitney U and η² for Kruskal-Wallis) to two decimal places.

Statistically significant p‑values were already highlighted in bold in the previous submission to support rapid interpretation.

Clear table legends explaining all abbreviations and statistical tests were included in the previous submission, and these have now been updated to incorporate the effect size measures as well.

Terminology and consistency

14. Several key concepts, including cultural awareness, cultural competence, person-centred care, and people of colour appear throughout the manuscript but are not always defined consistently or used with the same meaning. The authors should define each of these terms clearly and precisely on first use and then apply them consistently throughout the manuscript. This is particularly important for an international readership, where these terms may carry different connotations across healthcare, education, and research contexts.

Authors response: We have added clear definitions of key terms -including cultural awareness, cultural competence and, person‑centred care - at first use in the Introduction. We also reviewed the full manuscript to ensure these terms are applied consistently throughout. These revisions improve clarity and support accessibility for an international readership.

Excluded domain

15. The Research Issues (RI) domain of the original 36-item CAS was excluded from this study on the basis that undergraduate nursing students have limited research experience. While this rationale is understandable, it is not elaborated upon in sufficient methodological detail. The authors should clarify whether the exclusion of this domain affects comparability of their total CAS scores with those reported in previous studies that used the full 36-item version, and should discuss whether any re-validation or adaptation of the modified 32-item version was undertaken or whether the existing psychometric properties of the full scale can reasonably be assumed to apply to the modified version used here.

Authors response: Thank you for this comment. We have now elaborated clearly in the Methods section on the exclusion of the RI domain. Specifically, we explain that the RI items focus on research engagement and decision making processes that are not applicable to undergraduate nursing students, who typically have limited research experience and at this stage of their programme, and have not yet completed thesis or dissertation work.

This decision was also reviewed as part of the face validity assessment conducted by five expert reviewers (three senior nursing lecturers and two clinical tutors), who confirmed that the RI domain was not relevant for this population. As a result, the RI domain was removed, and the final instrument used in this study comprised 32 items. This rationale is now detailed in the Instrument and Variables subsection (page 8).

We also acknowledge that this modification has implications for comparability with previous studies using the full 36 item CAS. Specifically, the exclusion of the RI domain limits comparability with studies reporting total CAS scores based on all five original domains; however, comparability across the four retained subscales remains intact, as no items within these domains were removed or modified. This clarification has been added to the Limitations section (page 19).

We have also calculated and reported Cronbach’s alpha coefficients for the total scale and each of the four subscales, and we comment on these values in relation to findings from existing studies (see Instrument and Variables subsection, page 8).

Additionally, we have added a limitation acknowledging that the removal of the RI domain may have influenced the psychometric properties of the modified CAS, particularly construct validity, as a full validation was not feasible within the scope of this study (see Limitations subsection, page 19).

Language and style

16. Overall, the manuscript is clearly written and accessible. A careful proofread or light language editing pass to address minor typographical errors, grammatical inconsistencies, and formatting irregularities would enhance the overall professionalism and readability of the manuscript. This does not require major rewriting, but attention to detail at this level is expected for publication in a peer-reviewed journal.

Authors response: Thank you. The manuscript has been revised for typographical errors, grammatical inconsistencies, and formatting.

Overall Assessment

Overall, this manuscript addresses an important and timely topic, providing valuable insight into cultural awareness among undergraduate nursing students in Ireland and highlighting meaningful equity and inclusion issues within nursing education. The study is generally well structured, uses an appropriate and validated instrument, and produces findings that are broadly consistent with the existing international literature. However, several methodological concerns require substantive attention before the manuscript can be recommended for publication. These include the need to acknowledge the cross-sectional design's inability to establish causality, address potential self-report and social desirability bias, provide a more thorough discussion of non-response bias, justify the exclusion of the RI domain and its implications for comparability, consider multivariate analysis to account for confounding variables, and ensure that all interpretive claims are appropriately hedged and aligned with the study's empirical evidence. The treatment of ethnicity categorisation also requires stronger methodological justification. With these revisions, the paper has good potential to make a meaningful contribution to the literature on cultural competence and nursing education, and to inform curriculum development and institutional policy in the Irish and broader international nursing education context.

Authors response: Thank you very much for the feedback.

Attachment Submitted filename: Response to Reviewers.docx

# Editor report: Decision Letter 1

**DOI:** 10.1371/journal.pone.0359851.r003

**Authors:** Suad J Ghaben

9 Sep 2026

Dear Dr.  Hammoud,

Thank you for submitting your manuscript to PLOS One. After careful consideration, we feel that it has merit but does not fully meet PLOS One’s publication criteria as it currently stands. Therefore, we invite you to submit a revised version of the manuscript that addresses the points raised during the review process.

==============================

I invite you to respond to the editor’s comments and revise your manuscript.

1. The Subscale reliability is below acceptable thresholds. CA (α = 0.59) and BCI (α = 0.55) fall below the conventional 0.70 cutoff (some literature accepts a 0.60 threshold). The authors acknowledge this honestly and attribute it to short subscales, which is acceptable; however, this is not just a limitation to disclose; it means any non-significant finding on CA/BCI is uninterpretable (could be a true null or could be attenuation from unreliable measurement).
  This point should be explicitly flagged in the Results or Discussion sections, particularly where the subscales are interpreted as if the null findings were essentially meaningful. For example, language such as “differences… not statistically significant” in the CA/BCI comparisons should be accompanied by acknowledgment that measurement unreliability may provide an alternative explanation for the null results.

==============================

Please submit your revised manuscript by Oct 24 2026 11:59PM. If you will need more time than this to complete your revisions, please reply to this message or contact the journal office at plosone@plos.org. When you're ready to submit your revision, log on to https://www.editorialmanager.com/pone/ and select the 'Submissions Needing Revision' folder to locate your manuscript file.

- A letter that responds to each point raised by the academic editor and reviewer(s). You should upload this letter as a separate file labeled 'Response to Reviewers'.
- A marked-up copy of your manuscript that highlights changes made to the original version. You should upload this as a separate file labeled 'Revised Manuscript with Track Changes'.
- An unmarked version of your revised paper without tracked changes. You should upload this as a separate file labeled 'Manuscript'.

If you would like to make changes to your financial disclosure, please include your updated statement in your cover letter. Guidelines for resubmitting your figure files are available below the reviewer comments at the end of this letter.

If applicable, we recommend that you deposit your laboratory protocols in protocols.io to enhance the reproducibility of your results. Protocols.io assigns your protocol its own identifier (DOI) so that it can be cited independently in the future. For instructions see: https://journals.plos.org/plosone/s/submission-guidelines#loc-laboratory-protocols. Additionally, PLOS One offers an option for publishing peer-reviewed Lab Protocol articles, which describe protocols hosted on protocols.io. Read more information on sharing protocols at https://plos.org/protocols?utm_medium=editorial-email&utm_source=authorletters&utm_campaign=protocols.

As the corresponding author, your ORCID iD is verified in the submission system and will appear in the published article. PLOS supports the use of ORCID, and we encourage all coauthors to register for an ORCID iD and use it as well. Please encourage your coauthors to verify their ORCID iD within the submission system before final acceptance, as unverified ORCID iDs will not appear in the published article. *Only*  the individual author can complete the verification step; PLOS staff *cannot*  verify ORCID iDs on behalf of authors.

We look forward to receiving your revised manuscript.

Kind regards,

Suad J Ghaben, PhD

Academic Editor

PLOS One

Journal Requirements:

1. If the reviewer comments include a recommendation to cite specific previously published works, please review and evaluate these publications to determine whether they are relevant and should be cited. There is no requirement to cite these works unless the editor has indicated otherwise.

2. Please review your reference list to ensure that it is complete and correct. If you have cited papers that have been retracted, please include the rationale for doing so in the manuscript text, or remove these references and replace them with relevant current references. Any changes to the reference list should be mentioned in the rebuttal letter that accompanies your revised manuscript. If you need to cite a retracted article, indicate the article’s retracted status in the References list and also include a citation and full reference for the retraction notice.

[Note: HTML markup is below. Please do not edit.]

[NOTE: If reviewer comments were submitted as an attachment file, they will be attached to this email and accessible via the submission site. Please log into your account, locate the manuscript record, and check for the action link "View Attachments". If this link does not appear, there are no attachment files.]

To ensure your figures meet our technical requirements, please review our figure guidelines: https://journals.plos.org/plosone/s/figures

You may also use PLOS’s free figure tool, NAAS, to help you prepare publication quality figures: https://journals.plos.org/plosone/s/figures#loc-tools-for-figure-preparation.

NAAS will assess whether your figures meet our technical requirements by comparing each figure against our figure specifications.

# Author comment: Author response to Decision Letter 2

**DOI:** 10.1371/journal.pone.0359851.r004

14 Sep 2026

Dear Editor,

Thank you for your time in handling our manuscript. We have revised the manuscript in response to your comments and have highlighted the changes in the paper as requested. We have also provided detailed response to your comments. Please see our response below.

Editor’s Comments

1. The Subscale reliability is below acceptable thresholds. CA (α = 0.59) and BCI (α = 0.55) fall below the conventional 0.70 cutoff (some literature accepts a 0.60 threshold). The authors acknowledge this honestly and attribute it to short subscales, which is acceptable; however, this is not just a limitation to disclose; it means any non-significant finding on CA/BCI is uninterpretable (could be a true null or could be attenuation from unreliable measurement).

This point should be explicitly flagged in the Results or Discussion sections, particularly where the subscales are interpreted as if the null findings were essentially meaningful. For example, language such as “differences… not statistically significant” in the CA/BCI comparisons should be accompanied by acknowledgment that measurement unreliability may provide an alternative explanation for the null results.

Thank you for this comment. We have added a sentence to the Discussion section where we previously referred to non‑significant differences across the four subscale scores between students born in Ireland and those born outside Ireland, explicitly acknowledging the low reliability of the CA and BCI subscales.

“Given the lower reliability of the CA and BCI subscales, these non‑significant results should be interpreted cautiously, as limited measurement precision may obscure true differences.”

This has been added to the second paragraph of the Discussion and highlighted in green. As noted, this limitation was already addressed in the Strengths and Limitations section, so no further changes were required there. In the Conclusion, we only drew on statistically significant findings; therefore, no revisions were necessary in that section either.

Attachment Submitted filename: Response_to_Reviewers_auresp_2.docx

# Editor report: Decision Letter 2

**DOI:** 10.1371/journal.pone.0359851.r005

**Authors:** Suad J Ghaben

21 Sep 2026

Assessing Cultural Awareness and Competency in Nursing Education: A Cross-sectional Study of Undergraduate Nursing Students in Ireland

PONE-D-26-11506R2

Dear Dr. Hammoud,

We’re pleased to inform you that your manuscript has been judged scientifically suitable for publication and will be formally accepted for publication once it meets all outstanding technical requirements.

Within one week, you’ll receive an e-mail detailing the required amendments. When these have been addressed, you’ll receive a formal acceptance letter and your manuscript will be scheduled for publication.

An invoice will be generated when your article is formally accepted. Please note, if your institution has a publishing partnership with PLOS and your article meets the relevant criteria, all or part of your publication costs will be covered. Please make sure your user information is up-to-date by logging into Editorial Manager at Editorial Manager® and clicking the ‘Update My Information' link at the top of the page. For questions related to billing, please contact billing support.

If your institution or institutions have a press office, please notify them about your upcoming paper to help maximize its impact. If they’ll be preparing press materials, please inform our press team as soon as possible -- no later than 48 hours after receiving the formal acceptance. Your manuscript will remain under strict press embargo until 2 pm Eastern Time on the date of publication. For more information, please contact onepress@plos.org.

Kind regards,

Suad J Ghaben, PhD

Academic Editor

PLOS One

Additional Editor Comments (optional):

Reviewers' comments:

# Editor report: Acceptance letter

**DOI:** 10.1371/journal.pone.0359851.r006

**Authors:** Suad J Ghaben

PONE-D-26-11506R2

PLOS One

Dear Dr. Hammoud,

I'm pleased to inform you that your manuscript has been deemed suitable for publication in PLOS One. Congratulations! Your manuscript is now being handed over to our production team.

At this stage, our production department will prepare your paper for publication. This includes ensuring the following:

* All references, tables, and figures are properly cited

* All relevant supporting information is included in the manuscript submission,

* There are no issues that prevent the paper from being properly typeset

You will receive further instructions from the production team, including instructions on how to review your proof when it is ready. Please keep in mind that we are working through a large volume of accepted articles, so please give us a few days to review your paper and let you know the next and final steps.

Lastly, if your institution or institutions have a press office, please let them know about your upcoming paper now to help maximize its impact. If they'll be preparing press materials, please inform our press team within the next 48 hours. Your manuscript will remain under strict press embargo until 2 pm Eastern Time on the date of publication. For more information, please contact onepress@plos.org.

You will receive an invoice from PLOS for your publication fee after your manuscript has reached the completed accept phase. If you receive an email requesting payment before acceptance or for any other service, this may be a phishing scheme. Learn how to identify phishing emails and protect your accounts at https://explore.plos.org/phishing.

If we can help with anything else, please email us at customercare@plos.org.

Thank you for submitting your work to PLOS One and supporting open access.

Kind regards,

PLOS One Editorial Office Staff

on behalf of

Dr. Suad J Ghaben

Academic Editor

PLOS One
